# Correction: Urinary type IV collagen excretion is involved in the decline in estimated glomerular filtration rate in the Japanese general population without diabetes: A 5-year observational study

**DOI:** 10.1371/journal.pone.0199401

**Published:** 2018-06-21

**Authors:** The PLOS ONE Staff

## Notice of republication

This article was republished on June 5, 2018 to correct errors that were introduced during the typesetting process. The third, fourth, and fifth sentence under the subheading “Abnormal U-Col4CR was a significant independent risk factor for 10% of eGFR change per year in group 4” in the Results section were incorrectly included in the Table 4 caption. The publisher apologizes for the error. Please download this article again to view the corrected version. The originally published, uncorrected article and the republished, corrected article are provided here for reference.

## Supporting information

S1 File Originally published, uncorrected article. (PDF)

S2 File Republished, corrected article. (PDF)
