# Supplementary material for: Correction: Urinary type IV collagen excretion is involved in the decline in estimated glomerular filtration rate in the Japanese general population without diabetes: A 5-year observational study
Source: PLoS One. 2018 Jun 21;13(6):e0199401. doi: 10.1371/journal.pone.0199401 (PMC6013228; doi:10.1371/journal.pone.0199401)
Supplement: S2 File — (PDF) [file pone.0199401.s002.pdf]

RESEARCH ARTICLE

Urinary type IV collagen excretion is involved in the decline in estimated glomerular filtration rate in the Japanese general population without diabetes: A 5-year observational study

Fumi Kishi[1], Kojiro Nagai[1]*, Norimichi Takamatsu[2], Tatsuya Tominaga[3], Masanori Tamaki[1], Eriko Shibata[1], Taichi Murakami[1], Seiji Kishi[1], Hideharu Abe[1], Yasuhiko Koezuka[4], Naoto Minagawa[4], Go Ichien[4], Toshio Doi[1]

1 Department of Nephrology, Institute of Biomedical Sciences, Tokushima University Graduate School, Tokushima, Japan, **2** Department of Kidney Disease (Dialysis & Transplantation), Kawashima Hospital, Tokushima, Japan, **3** Department of Chronomedicine, Institute of Biomedical Sciences, Tokushima University Graduate School, Tokushima, Japan, **4** HuBit Genomix Inc., Tokyo, Japan

* knagai@tokushima-u.ac.jp

OPEN ACCESS

Citation: Kishi F, Nagai K, Takamatsu N, Tominaga T, Tamaki M, Shibata E, et al. (2018) Urinary type IV collagen excretion is involved in the decline in estimated glomerular filtration rate in the Japanese general population without diabetes: A 5-year observational study. PLoS ONE 13(4): e0195523. https://doi.org/10.1371/journal.pone.0195523

Editor: Tatsuo Shimosawa, The University of Tokyo, JAPAN

Received: November 16, 2017

Accepted: March 23, 2018

Published: April 6, 2018

Copyright: © 2018 Kishi et al. This is an open access article distributed under the terms of the Creative Commons Attribution License, which permits unrestricted use, distribution, and reproduction in any medium, provided the original author and source are credited.

Data Availability Statement: All relevant data are within the paper and its Supporting Information files.

Funding: The authors received no specific funding for this work. HuBit Genomix Inc. provided support in the form of salaries for authors YK, NM and GI but did not have any additional role in the study design, data collection and analysis, decision to publish, or preparation of the manuscript. The

Abstract

Urinary type IV collagen (U-Col4) and albumin excretion is evaluated to monitor the development of diabetic kidney disease. However, U-Col4 excretion in the general population without diabetes has not yet been fully elucidated. In this study, 1067 participants without diabetes and with urinary albumin-creatinine ratio <300 mg/gCr (normo- or microalbuminuria) who underwent an annual health examination in 2004 were enrolled and observed for 5 years. They were divided according to the amount of U-Col4 or urinary albumin excreted. The decline in estimated glomerular filtration rate (eGFR) was calculated. In participants with eGFR $\geq$80 mL/min, abnormal U-Col4 excretion was indicated as a significant independent risk factor for 10% eGFR change per year, which is one of the prognostic factors for the development of end-stage kidney disease. Moreover, in contrast to urinary albumin excretion, U-Col4 excretion was not related to age or kidney function, suggesting that some individuals with abnormal U-Col4 excretion can have an independent hidden risk for the development of kidney dysfunction. In conclusion, it is important to measure U-Col4 excretion in the general population without diabetes to determine changes in renal features in every individual and help detect future complications such as diabetic kidney disease. If U-Col4 excretion is abnormal, kidney manifestation should be carefully followed up, even if the kidney function and urinalysis findings are normal.

Introduction

Urinary type IV collagen (U-Col4) and albumin excretion is used to monitor the development of diabetic nephropathy and is associated with the disease progression [1–4]. Microalbuminuria was also included in the definition of generic chronic kidney disease (CKD) in the

specific roles of these authors are articulated in the 'author contributions' section.

Competing interests: The authors declare that co-author Hideharu Abe is a PLOS ONE Editorial board member. The authors also declare that co-authors Naoto Minagawa, Yasuhiko Koezuka and Go Ichien belong to HuBit Genomix Inc. and are employed by a commercial company (HuBit Genomix Inc.). This does not alter the authors' adherence to all the PLOS ONE policies on sharing data and materials.

original classification schema of the Kidney Disease Outcomes Quality Initiative (KDOQI) of the National Kidney Foundation (NKF) in 2002 [5]. Regardless of the underlying mechanisms and interpretation of the pathophysiologic meaning of albuminuria, important associations exist between the quantities of albumin excreted in the urine and progressive CKD and cardiovascular events [6]. In contrast, U-Col4 excretion in patients with nondiabetic kidney disease and the general population has not yet been fully elucidated. Furumatsu et al. evaluated U-Col4 in nondiabetic kidney disease and reported that elevation of U-Col4 was distinctively observed in the patients with membranous nephropathy and anti-neutrophil cytoplasmic antibody-associated glomerulonephritis [7]. We previously examined the U-Col4-creatinine ratio (U-Col4CR) in the general population. The mean U-Col4CR and urinary albumin-creatinine ratio (ACR) values increased in individuals with estimated glomerular filtration rate (eGFR) $<$60 mL/min/1.73m^2 compared to that in individuals with eGFR of 60–89 mL/min/1.73m^2 and $\geq$90 mL/min/1.73m^2. However, the incidence rate of abnormal U-Col4CR was around 15–20%, and was similar among individuals with eGFR $<$60 mL/min/1.73m^2, 60–89 mL/min/1.73m^2, and $\geq$90 mL/min/1.73m^2, whereas the incidence rate of high ACR increased significantly in individuals with low eGFR values [8]. To our best knowledge, no study has investigated whether abnormal U-Col4CR in the general population is associated with a decline in the eGFR.

Therefore, in this study, we examined the relationship between U-Col4CR and ACR and eGFR change to clarify their role in the progression of kidney dysfunction in the general population.

Materials and methods

Ethical approval

This study was approved by the Ethics Committee of the Arita-cho, HuBit genomics, Inc. and was performed in compliance with the Helsinki Declaration. Written informed consent was obtained from all participants.

Design and subjects

In 2004, 1376 participants in the town of Arita in Japan were registered who underwent an annual health examination and gave us written informed consent. Their health examinations were continued annually. We excluded the participants: (i) if they did not revisit their annual health examinations until 2009 (n = 198) because we could not calculate their declines in eGFR: (ii) if they exhibited ACR $\geq$300 mg/gCr (macroalbuminuria) (n = 7) because the number of participants with macroalbuminuria was not enough to analyze the prognosis statistically: (iii) if they had taken diabetes medications and/or their HbA1c (NGSP) was $\geq$6.5% and/or non-fasting blood glucose level was $\geq$200 mg/dL (n = 104) because we focused on the prognosis of participants without diabetes [9]. Finally, 1067 participants were enrolled in this study (Fig 1). Of the 1067 participants, 335 got an annual health examination once (290 in 2005, 18 in 2006, 17 in 2007, 2 in 2008, 8 in 2009), 152 got twice, 223 got 3 times, 155 got 4 times and 202 got 5 times for the period of 5 year. The follow-up year was 3.1 ± 1.6 (mean ± SD) years.

As described previously [8], this study is a community-based survey that consisted of a self-administered questionnaire on lifestyle, medical histories, anthropometrical measurements, and collections of blood and urine specimens from participants. All relevant data derived from their annual health examinations can be found in S1 File.

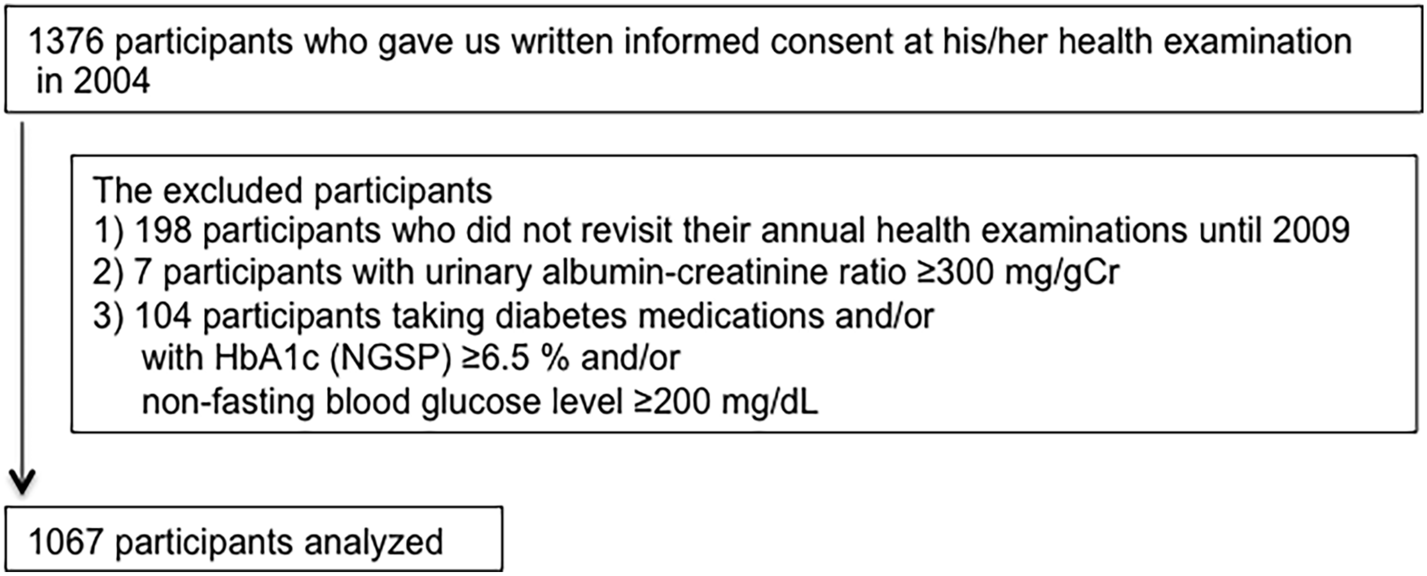

Fig 1. Study participants.

https://doi.org/10.1371/journal.pone.0195523.g001

Questionnaires, blood pressure measurement and body mass index

Participants used a self-report questionnaire to document medical history and lifestyle. They answered whether they had ever been treated with any of the followings: diabetes mellitus, hypertension, and hyperlipidemia. They answered alcohol intake (drinker or non-drinker), smoking habit (smoker or non-smoker). Systolic and diastolic blood pressures were measured by using an automatic oscillometric method using HEM-780 (OMRON Corporation, Kyoto, Japan) in the sitting position after at least 5 minutes rest. Measurement was performed twice, and the mean value was used for statistical analysis. Body mass index was calculated by dividing weight (kg) by height (m) squared.

Laboratory methods and estimation of glomerular filtration rate

Blood samples were taken from all participants for the assessment of laboratory parameters once during a health examination. Serum creatinine (Cr) concentration was measured by the Jaffe method using an AU5431 auto analyzer (Olympus Corporation, Tokyo, Japan). The value of serum Cr was corrected by subtracting 0.2 mg/dL to that measured by the enzymatic method [10]. eGFR of each participant was calculated from corrected serum Cr and age by using the 3-variable Japanese equation as follows [11].

$$\mathrm{eGFR}\ (\mathrm{mL/min/1.73m^2}) = 194\ \mathrm{X}\ \mathrm{Age}^{-0.287}\ \mathrm{X}\ \mathrm{Cr}\ (\mathrm{mg/dL})^{-1.094}\ (\mathrm{X}\ 0.739\ \mathrm{if\ female}).$$

In order to increase the accuracy of individual eGFR change, eGFR data shown in this analysis are the eGFR indexed by body surface area calculated by the Du Bois formula [12]. To calculate a decline in eGFR per year, a simple linear regression line was estimated by the least squares method using all eGFR measurements for each participant (2 to 6 samples, mean: 3.75 samples). The calculated eGFR in each year was determined using the linear regression line. Last eGFR was defined as the latest calculated eGFR during the observation period. If the latest calculated eGFR was below 0 mL/min, the calculated eGFR before the latest calculated eGFR

was used as last eGFR (n = 11). %eGFR change per year was calculated as follows [9].

$$\%\text{eGFR change per year } (\%) = (\text{last eGFR} - \text{eGFR in } 2004)/\ \text{eGFR in } 2004/\ (\text{the year of last eGFR} -\ 2004)\ \text{X } 100.$$

HbA1c (the Japan Diabetes Society: JDS) was measured by high performance liquid chromatography. HbA1c (JDS) was converted to HbA1c (NGSP) according to the equation: HbA1c (NGSP) (%) = 1.02 X HbA1c (JDS) (%)+ 0.25 [13].

Urine samples were collected from participants at their homes and transported to the laboratory. Urinary Cr concentration was measured by the enzymatic method (Shino-Test Corporation, Tokyo, Japan). Urinary albumin concentration was measured by a turbidimetric immunoassay (Wako Diagnostics, Osaka, Japan). ACR was calculated as urinary albumin concentration divided by urinary Cr concentration. Microalbuminuria was defined as ACR of 30–300 mg/gCr [14]. U-Col4 level was measured by a one-step sandwich enzyme immunoassay using two monoclonal antibodies against the 7S domain and the triple helix domain of human placental type IV collagen (Daiichi Fine Chemical Co Ltd., Toyama, Japan) [15,16]. U-Col4CR was determined as U-Col4 concentration divided by urinary Cr concentration. The normal cut-off range of U-Col4CR was <8.2 μg/gCr and <9.4 μg/gCr for males and females, respectively, determined by using the urine samples of 442 healthy adults (age, 20–70 years) who yielded normal results on both physical examination and laboratory analyses as shown in our previous report [8].

Statistical analysis

The characteristics of multiple groups were analyzed using one-way factorial ANOVA or Kruskal-Wallis test. The declines in eGFR among eGFR groups were compared by using Dunnett's test. The characteristics of two groups were analyzed using student's t-test or Wilcoxon rank sum test. F-test was used for comparing the factors of total deviation. Categorical data were compared by Fisher's exact test. Risk factors for 10% eGFR change per year were evaluated by a multivariate logistic regression analysis using demographic and clinical characteristics. If there were strong correlations (r_s >0.5) between characteristics, one representative variable was chosen to the analysis, that is ALT and hemoglobin, which were correlated with AST, γ-GTP and red blood cell count, hematocrit, respectively (data not shown). Non-fasting blood glucose level was deleted because of the presence of HbA1c. Serum Cr concentration was deleted because participants were already divided based on eGFR. Consequently, U-Col4CR, ACR (normoalbuminuria vs microalbuminuria or the actual value of ACR), age, gender, body mass index, drinker, smoker, systolic blood pressure, diastolic blood pressure, HbA1c, ALT, total cholesterol, triglyceride, HDL-cholesterol and hemoglobin were used for the analysis. Kaplan-Meier curves were plotted to estimate the prognostic value of abnormal U-Col4CR (vs. normal U-Col4CR) or microalbuminuria (vs. normoalbuminuria) for a decline in eGFR. The event was defined as the first occurrence of 10% eGFR change per year (90, 80, 70, 60, 50% of eGFR in 2004 in 1, 2, 3, 4, 5 years, respectively) in the actual eGFR value of participants with 10% eGFR change per year. Log-Rank test was used to compare the cumulative incidences of two groups. All values are expressed as mean ± SD. Statistical analysis was performed using Windows SAS 9.4 (SAS Institute Inc., Cary, NC, USA.). A statistical significance was defined by *P* less than 0.05.

Results

Study participants

A total of 1067 participants aged 20 to 91 years (mean ± SD, 61.9 ± 13.6 years) were included in this study, with 677 participants (63.4%) being women. Of all participants, 522 (48.9%) were

drinkers and 134 (12.6%) were smokers. Their mean age, gender distribution, and drinking and smoking habits were similar with those in recent large Japanese community-based cohort studies [17–19]. U-Col4CR ranged from 0.9 to 47.7 μg/gCr (6.19 ± 3.52 μg/gCr); 149 participants (14.0%) had abnormal U-Col4CR. ACR varied from 0.7 to 291.9 mg/gCr (13.66 ± 21.91 mg/gCr); 77 participants (7.2%) had microalbuminuria. Systolic and diastolic blood pressures were 130.2 ± 21.0 and 78.0 ± 11.0 mmHg, respectively. HbA1c level was 5.45 ± 0.39%. Of the participants, 253 (23.7%) and 95 (8.9%) underwent treatment for hypertension and hyperlipidemia, respectively.

Decline in eGFR in participants without diabetes with normo- or microalbuminuria

The decline in eGFR was evaluated in this study. The participants were divided into 4 groups based on eGFR quartiles (group 1: <58.2 mL/min, group 2: 58.2–<68.1 mL/min, group 3: 68.1–<80 mL/min, group 4: ≥80 mL/min); participants with the highest eGFR (≥80 mL/min) had a faster decline than those with eGFR <68.1 mL/min (Fig 2). Our results were consistent with those of the previous report by Imai et al., which observed a faster decline in eGFR in the aged group (≥60 years) with eGFR ≥70 mL/min/1.73m^2 than in those with eGFR of 60–69 mL/min/1.73m^2 [20].

Distribution of participants with abnormal U-Col4CR or high ACR (microalbuminuria) in each eGFR group

To examine the effect of abnormal U-Col4CR or high ACR (microalbuminuria) on a decline in eGFR, the participants in each eGFR group were analyzed. The lower eGFR group included more older and female participants with higher ACR and systolic blood pressure (Table 1). Levels of HbA1c, non-fasting blood glucose, total cholesterol, triglyceride were higher, and levels of ALT, hemoglobin were lower in the lower eGFR group. The number of participants

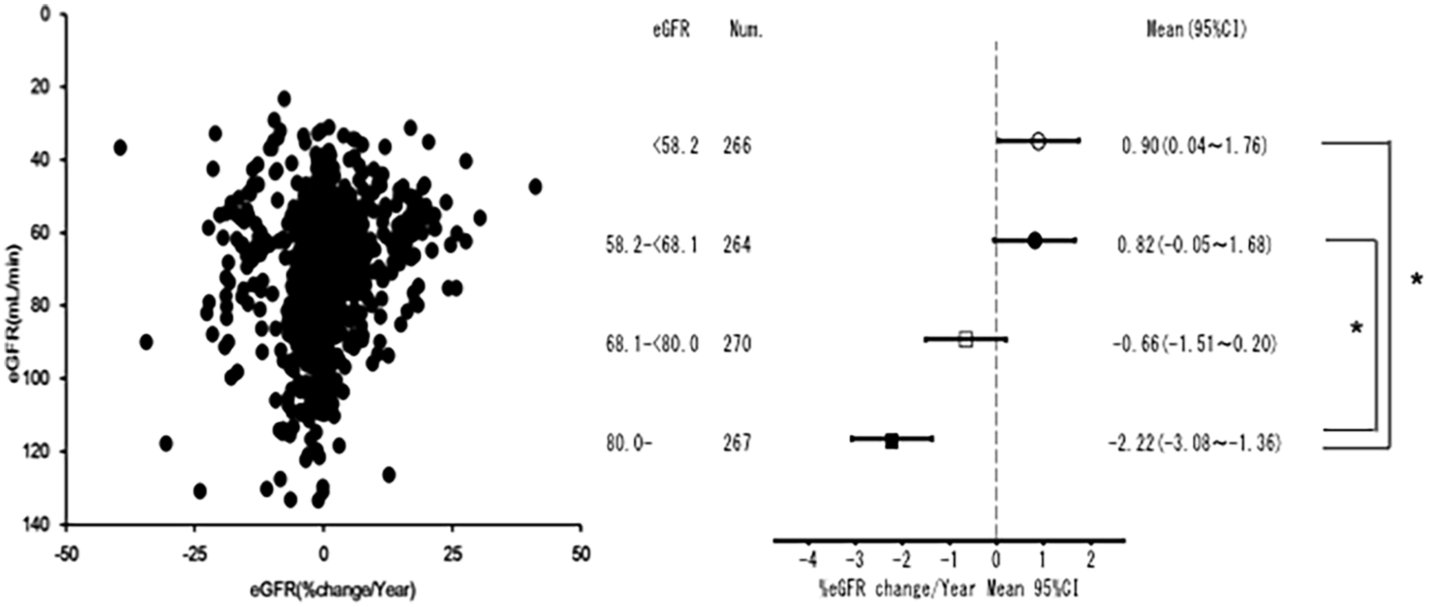

Fig 2. Decline in eGFR in participants without diabetes with normo- or microalbuminuria. Participants with the highest eGFR (≥80 mL/min) had a faster decline in eGFR than those with eGFR <68.1 mL/min. Num.: Number. 95%CI: 95% confidence interval. *P <0.01.

https://doi.org/10.1371/journal.pone.0195523.g002

Table 1. Distribution of participants with abnormal urinary type IV collagen-creatinine ratio or microalbuminuria in each eGFR group.

eGFR group	1 (<58.2)	2 (<68.1)	3 (<80.0)	4 (≥80.0)
Number, n	266	264	270	267
U-Col4CR, μg/gCr	6.35 ± 4.16	5.98 ± 3.11	6.16 ± 3.23	6.28 ± 3.49
N, Normal, n (%) A, Abnormal, n (%)	228 (85.7%) 38 (14.3%)	230 (87.1%) 34 (12.9%)	233 (86.3%) 37 (13.7%)	227 (85.0%) 40 (15.0%)
ACR, mg/gCr*	18.2 ± 31.6	13.3 ± 21.3	11.1± 9.5	12.1± 19.0
L, Normo, n (%) M, Micro, n (%)	237 (89.1%) 29 (10.9%)	245 (92.8%) 19 (7.2%)	256 (94.8%) 14 (5.2%)	252 (94.4%) 15 (5.6%)
Age, years*	70.4 ± 8.4	65.4 ± 10.5	60.6 ± 11.9	51.2 ± 14.5
Female, %*	195 (73.3%)	171 (64.8%)	160 (59.3%)	151 (56.6%)
BMI, kg/m^2	22.13 ± 3.09	22.41 ± 2.71	22.44 ± 2.72	22.65 ± 3.19
Drinker, n (%)*	92 (34.6%)	113 (42.8%)	142 (52.6%)	175 (65.5%)
Smoker, n (%)*	15 (5.6%)	19 (7.2%)	41 (15.2%)	59 (22.1%)
SBP (mmHg)*	134.7 ± 21.3	131.1 ± 20.8	129.7 ± 19.6	125.4 ± 21.4
DBP (mmHg)	78.8 ± 10.9	78.4 ± 10.3	78.1 ± 11.1	76.9 ± 11.5
HbA1c (%)*	5.55 ± 0.36	5.50 ± 0.37	5.42 ± 0.39	5.33 ± 0.41
NFBG (mg/dL)*	85.5 ± 13.3	83.3 ± 9.5	84.2 ± 12.6	82.2 ± 10.8
ALT (IU/L)**	18.9 ± 8.6	20.8 ± 11.7	22.3 ± 13.2	22.2 ± 13.5
T-chol (mg/dL)*	202.7 ± 29.8	200.3 ± 30.8	195.3 ± 34.8	189.1 ± 31.4
HDL-C (mg/dL)	54.6 ± 11.5	54.0 ± 12.1	55.5 ± 13.3	54.8 ± 13.0
TG (mg/dL)*	103.7 ± 50.0	102.0 ± 56.6	103.5 ± 65.9	93.6 ± 56.5
Cre (mg/dL)*	0.81 ± 0.20	0.70 ± 0.14	0.65 ± 0.14	0.58 ± 0.14
Hb (g/dL)*	13.01 ± 1.33	13.39 ± 1.24	13.57 ± 1.55	13.60 ± 1.76
HT. treat.*	102 (38.3%)	70 (26.5%)	42 (15.6%)	39 (14.6%)
HL. treat.*	37 (13.9%)	30 (11.4%)	18 (6.7%)	10 (3.7%)

eGFR: Estimated glomerular filtration rate. Group 1: <58.2 mL/min. Group 2: 58.2-<68.1 mL/min. Group 3: 68.1-<80.0 mL/min. Group 4: ≥80.0 mL/min. U-Col4CR: Urinary type IV collagen-creatinine ratio. N, Normal: Normal urinary type IV collagen-creatinine ratio. A, Abnormal: Abnormal urinary type IV collagen-creatinine ratio. ACR: Urinary albumin-creatinine ratio. L, Normo: Normoalbuminuria. M, Micro: Microalbuminuria. BMI: Body mass index. SBP: Systolic blood pressure. DBP: Diastolic blood pressure. NFBG: Non-fasting blood glucose level. T-chol: Total cholesterol. HDL-C: HDL-cholesterol. TG: Triglyceride. Cre: Creatinine. Hb: Hemoglobin. HT. treat.: Participants underwent treatment for hypertension. HL. treat.: Participants underwent treatment for hyperlipidemia.

*: $P < 0.01$.

**: $P < 0.05$

https://doi.org/10.1371/journal.pone.0195523.t001

undergoing treatment for hypertension or hyperlipidemia was higher in the lower eGFR group. Consequently, the participants in the lower eGFR group experienced more complications than those in the higher eGFR group. It is notable that both the mean value of U-Col4CR and the number of participants with abnormal U-Col4CR showed no difference among the groups.

Characteristics of participants with or without 10% of eGFR change per year in each group

To determine the risk factors for a decline in eGFR, we divided participants in the 4 groups mentioned previously according to the %eGFR change per year faster or slower than 10%, because 30% eGFR change over 2 or 3 years was suggested as one of the prognostic factors for the development of end-stage kidney disease [9, 21–24], and compared demographic and clinical variables between participants with the change per year of eGFR by 10% and those without the change (Tables 2 and 3). More participants with abnormal U-Col4CR were included in those with the change than those without the change in group 4. ACR was significantly higher in participants with the change in group 3 and 4. There were several other variables such as old

Table 2. Characteristics of participants with or without 10% of eGFR change per year in each group.

eGFR group	1 (<58.2)		2 (58.2-<68.1)	
%eGFR change per year	≤-10%	>-10%	≤-10%	>-10%
Number, n	25	241	14	250
U-Col4CR, μg/gCr	6.96 ± 3.37	6.28 ± 4.24	7.25 ± 5.77	5.91 ± 2.90
N,Normal, n (%) A,Abnormal,n(%)	19 (76.0%) 6 (24.0%)	209 (86.7%) 32 (13.3%)	11 (78.6%) 3 (21.4%)	219 (87.6%) 31 (12.4%)
ACR, mg/gCr	23.9 ± 33.1	17.6 ± 31.5	18.8 ±18.6	13.0 ± 21.4
L, Normo, n (%) M, Micro, n (%)	22 (88.0%) 3 (12.0%)	215 (89.2%) 26 (10.8%)	11 (78.6%) 3 (21.4%)	234 (93.6%) 16 (6.4%)
Age, years	78.6 ± 4.3	69.6 ± 8.3*	74.6 ± 15.7	64.9 ± 9.9**
Female, %	19 (76.0%)	176 (73.0%)	5 (35.7%)	166(66.4%)**
BMI, kg/m^2	21.58 ± 2.94	22.19 ± 3.10	20.79 ± 3.05	22.50 ±2.66**
Drinker, n (%)	5 (20.0%)	87 (36.1%)	6 (42.9%)	107 (42.8%)
Smoker, n (%)	2 (8.0%)	13 (5.4%)	1 (7.1%)	18 (7.2%)
SBP (mmHg)	147.6 ± 22.0	133.4 ± 20.8*	133.1 ± 22.1	131.0 ± 20.8
DBP (mmHg)	82.5 ± 13.5	78.4 ± 10.6	77.6 ± 7.0	78.5 ± 10.5
HbA1c (%)	5.64 ± 0.42	5.55 ± 0.36	5.48 ± 0.38	5.50 ± 0.37
NFBG (mg/dL)	83.8 ± 8.3	85.7 ± 13.7	84.4 ± 12.0	83.2 ± 9.4
ALT (IU/L)	21.4 ± 13.6	18.6 ± 7.8	19.1 ± 11.4	20.9 ± 11.8
T-chol (mg/dL)	190.1 ± 25.0	204.1 ± 30.0**	181.1 ± 31.6	201.3 ±30.5**
HDL-C (mg/dL)	52.4 ± 10.6	54.8 ± 11.6	53.9 ± 9.1	54.0 ± 12.2
TG (mg/dL)	111.9 ± 58.4	102.9 ± 49.1	80.8 ± 43.4	103.2 ± 57.1
Hb (g/dL)	12.54 ± 1.32	13.06 ± 1.33	13.44 ± 1.41	13.39 ± 1.23
HT. treat.	17 (68.0%)	85 (35.3%)*	4 (28.6%)	66 (26.4%)
HL. treat.	3 (12.0%)	34 (14.1%)	0 (0.0%)	30 (12.0%)

eGFR: Estimated glomerular filtration rate. Group 1: <58.2 mL/min. Group 2: 58.2-<68.1 mL/min. U-Col4CR: Urinary type IV collagen-creatinine ratio. N, Normal: Normal urinary type IV collagen-creatinine ratio. A, Abnormal: Abnormal urinary type IV collagen-creatinine ratio. ACR: Urinary albumin-creatinine ratio. L, Normo: Normoalbuminuria. M, Micro: Microalbuminuria. BMI: Body mass index. SBP: Systolic blood pressure. DBP: Diastolic blood pressure. NFBG: Non- fasting blood glucose level. T-chol: Total cholesterol. HDL-C: HDL-cholesterol. TG: Triglyceride. Hb: Hemoglobin. HT. treat.: Participants underwent treatment for hypertension. HL. treat.: Participants underwent treatment for hyperlipidemia.

*: $P < 0.01$.

**: $P < 0.05$.

https://doi.org/10.1371/journal.pone.0195523.t002

age (group 1,2,3) and high systolic blood pressure (group 1) related to participants with the change.

Abnormal U-Col4CR was a significant independent risk factor for 10% of eGFR change per year in group 4

Odds ratios of U-Col4CR and ACR for 10% of eGFR change per year were determined in participants with normo- or microalbuminuria in a multivariate logistic regression analysis. Abnormal U-Col4CR was a significant risk factor for 10% of eGFR change per year in group 4, even after adjustment for demographic and clinical characteristics, whereas microalbuminuria was not a significant risk factor in all groups (Table 4).

Abnormal U-Col4CR was also a significant risk factor for 10% of eGFR change per year, even after adjustment for the characteristics in participants with normoalbuminuria in group 4 only (data not shown). If the odds ratio of the actual value of ACR was determined in the same analysis, abnormal U-Col4CR was still a significant risk factor for 10% of eGFR change per year in group 4. ACR was revealed as a significant risk factor for 10% of eGFR change per year in group 3, even after adjustment for the characteristics (Table 5).

Table 3. Characteristics of participants with or without 10% of eGFR change per year in each group.

eGFR group	3 (68.1-<80.0)		4 (≥80.0)	
%eGFR change per year	≤-10%	>-10%	≤-10%	>-10%
Number, n	13	257	15	252
U-Col4CR, μg/gCr	5.78 ± 2.62	6.18 ± 3.26	8.47 ± 4.75	6.15 ± 3.37**
N,Normal, n (%) A,Abnormal,n(%)	12 (92.3%) 1 (7.7%)	221 (86.0%) 36 (14.0%)	8 (53.3%) 7 (46.7%)	219 (86.9%)* 33 (13.1%)
ACR, mg/gCr	16.0 ± 9.5	10.9 ± 9.4*	18.6 ± 16.3	11.7 ± 19.1**
L, Normo, n (%) M, Micro, n (%)	12 (92.3%) 1 (7.7%)	244 (94.9%) 13 (5.1%)	13 (86.7%) 2 (13.3%)	239 (94.8%) 13 (5.2%)
Age, years	71.5 ± 7.8	60.0 ± 11.8*	52.8 ± 19.3	51.1 ± 14.3
Female, %	6 (46.2%)	154 (59.9%)	10 (66.7%)	141 (56.0%)
BMI, kg/m^2	22.05 ± 2.53	22.46 ± 2.73	22.69 ± 2.88	22.64 ± 3.21
Drinker, n (%)	7 (53.8%)	135 (52.5%)	11 (73.3%)	164 (65.1%)
Smoker, n (%)	3 (23.1%)	38 (14.8%)	5 (33.3%)	54 (21.4%)
SBP (mmHg)	136.0 ± 14.7	129.4 ± 19.8	133.4 ± 26.9	125.0 ± 21.0
DBP (mmHg)	79.8 ± 10.5	78.0 ± 11.2	78.5 ± 11.5	76.8 ± 11.5
HbA1c (%)	5.35 ± 0.42	5.42 ± 0.39	5.21 ± 0.50	5.34 ± 0.41
NFBG (mg/dL)	82.8 ± 10.1	84.3 ± 12.7	79.5 ± 7.2	82.4 ± 11.0
ALT (IU/L)	15.4 ± 4.5	22.6 ±13.4**	21.3 ± 13.4	22.3 ± 13.6
T-chol (mg/dL)	182.8 ± 25.5	196.0 ± 35.1	175.5 ± 26.9	189.9 ± 31.5
HDL-C (mg/dL)	51.0 ± 13.7	55.7 ± 13.2	49.2 ± 9.3	55.1 ± 13.1
TG (mg/dL)	100.2 ± 67.1	103.7 ± 66.0	100.5 ± 65.8	93.2 ± 56.0
Hb (g/dL)	13.09 ± 1.64	13.60 ± 1.55	13.90 ± 1.83	13.58 ± 1.76
HT. treat.	1 (7.7%)	41 (16.0%)	6 (40.0%)	33 (13.1%)**
HL. treat.	1 (7.7%)	17 (6.6%)	0 (0.0%)	10 (4.0%)

eGFR: Estimated glomerular filtration rate. Status 3: 68.1-<80.0 mL/min. Status 4: ≥80.0 mL/min. U-Col4CR: Urinary type IV collagen-creatinine ratio. N, Normal: Normal urinary type IV collagen-creatinine ratio. A, Abnormal: Abnormal urinary type IV collagen-creatinine ratio. ACR: Urinary albumin-creatinine ratio. L, Normo: Normoalbuminuria. M, Micro: Microalbuminuria. BMI: Body mass index. SBP: Systolic blood pressure. DBP: Diastolic blood pressure. NFBG: Non- fasting blood glucose level. T-chol: Total cholesterol. HDL-C: HDL-cholesterol. TG: Triglyceride. Hb: Hemoglobin. HT. treat.: Participants underwent treatment for hypertension. HL. treat.: Participants underwent treatment for hyperlipidemia.

*: $P < 0.01$.

**: $P < 0.05$.

https://doi.org/10.1371/journal.pone.0195523.t003

Kaplan-Meier curves to evaluate the prognostic value of U-Col4CR or ACR for 10% eGFR change per year

In each group, Kaplan-Meier curves were plotted to evaluate the prognostic value of U-Col4CR or ACR for 10% eGFR change per year. Abnormal U-Col4CR in group 4 and microalbuminuria in group 2 were statistically significant for the prediction of 10% eGFR change per year (Fig 3).

Discussion

In this study, we demonstrated that U-Col4CR was a significant independent risk factor for the decline in eGFR in the Japanese general population without diabetes who had ACR <300 mg/gCr and eGFR ≥80 mL/min. To our knowledge, this is the first report that showed the importance of U-Col4CR measurement even in the general population without diabetes.

A novel finding of this study is that abnormal U-Col4CR can be one of the prognostic factors for kidney deterioration in individuals without diabetes and apparent proteinuria with normal kidney function. The usefulness of U-Col4CR as well as ACR to monitor the

Table 4. Odds ratios of urinary type IV collagen-creatinine ratio (U-Col4CR) and urinary albumin-creatinine ratio (ACR) for 10% of eGFR change per year.

eGFR group	Variable	OR	95%CI	*P*	Adj. OR	95%CI	*P*
1 (<58.2)	U-Col4CR A (vs. N)	2.089	0.756–5.774	0.156	2.175	0.654–7.239	0.205
	ACR M (vs. L)	0.931	0.250–3.467	0.915	0.428	0.076–2.406	0.335
2 (<68.1)	U-Col4CR A (vs. N)	1.647	0.420–6.457	0.474	1.227	0.211–7.131	0.820
	ACR M (vs. L)	3.697	0.917–14.909	0.066	4.436	0.685–28.740	0.118
3 (<80.0)	U-Col4CR A (vs. N)	0.476	0.058–3.874	0.488	0.318	0.026–3.954	0.373
	ACR M (vs. L)	1.808	0.211–15.477	0.589	2.868	0.183–45.012	0.453
4 (≥80.0)	U-Col4CR A (vs. N)	5.516	1.787–17.024	0.003	7.086	1.640–30.611	0.009
	ACR M (vs. L)	1.331	0.241–7.351	0.743	1.623	0.226–11.650	0.623

OR: Odds ratio. Adj. OR: Adjusted odds ratio. 95%CI: 95% confidence interval. A: Abnormal urinary type IV collagen-creatinine ratio. N: Normal urinary type IV collagen-creatinine ratio. M: Microalbuminuria. L: Normoalbuminuria. The variables adjusted for: age, gender, body mass index, drinker, smoker, systolic blood pressure, diastolic blood pressure, HbA1c, ALT, total cholesterol, triglyceride, HDL-cholesterol, hemoglobin.

https://doi.org/10.1371/journal.pone.0195523.t004

development of diabetic nephropathy is well recognized. However, the importance of U-Col4CR in the general population has not been determined yet. We investigated the possible risk factors, including U-Col4CR and ACR, for the decline in eGFR in the general population without diabetes with normo- or microalbuminuria. Several reports have described the risk factors for kidney deterioration in patients with CKD, but few have investigated or discussed the eGFR change in individuals with eGFR ≥60 mL/min/1.73m^2 [9,20–24]. Therefore, we focused on the decline of eGFR in the general population without diabetes and apparent proteinuria with normal kidney function. Abnormal U-Col4CR was identified as a prognostic factor for the decline in eGFR in individuals with eGFR ≥80 mL/min (Tables 4 and 5, Fig 3).

Conversely, microalbuminuria was not a significant risk factor for the decline in eGFR in the general population without diabetes (Table 4). However, the definition of microalbuminuria as ≥30 mg/gCr may not be precise. This is consistent with those of other previous reports. Several studies have shown that ACR reference values have inherent racial disparities resulting from either albumin [25] or creatinine excretion [26]. Using the Caucasian data sets, KDOQI recommended ACR reference values of <17 mg/gCr for males and <25 mg/gCr for females [27]. Thus, we recognize the importance of ACR as well as U-Col4CR as one of the possible prognostic factors for the decline in eGFR in the general population without diabetes

Table 5. Odds ratios of urinary type IV collagen-creatinine ratio (U-Col4CR) and urinary albumin-creatinine ratio (ACR) for 10% of eGFR change per year in participants with normoalbuminuria only.

eGFR group	Variable	OR	95%CI	*P*	Adj. OR	95%CI	*P*
1 (<58.2)	U-Col4CR A (vs. N)	2.786	0.956–8.121	0.061	3.005	0.815–11.078	0.098
	ACR mg/gCr	1.056	0.994–1.122	0.077	1.013	0.937–1.094	0.752
2 (<68.1)	U-Col4CR A (vs. N)	0.704	0.086–5.735	0.743	0.842	0.074–9.552	0.890
	ACR mg/gCr	1.045	0.947–1.154	0.381	1.056	0.936–1.193	0.374
3 (<80.0)	U-Col4CR A (vs. N)	0.511	0.061–4.290	0.536	0.133	0.005–3.902	0.242
	ACR mg/gCr	1.132	1.044–1.229	0.003	1.197	1.049–1.367	0.008
4 (≥80.0)	U-Col4CR A (vs. N)	5.439	1.627–18.183	0.006	12.838	2.133–77.264	0.005
	ACR mg/gCr	1.105	1.020–1.196	0.015	1.101	0.992–1.223	0.071

OR: Odds ratio. Adj. OR: Adjusted odds ratio. 95%CI: 95% confidence interval. A: Abnormal urinary type IV collagen-creatinine ratio. N: Normal urinary type IV collagen-creatinine ratio. The variables adjusted for: age, gender, body mass index, drinker, smoker, systolic blood pressure, diastolic blood pressure, HbA1c, ALT, total cholesterol, triglyceride, HDL-cholesterol, hemoglobin.

https://doi.org/10.1371/journal.pone.0195523.t005

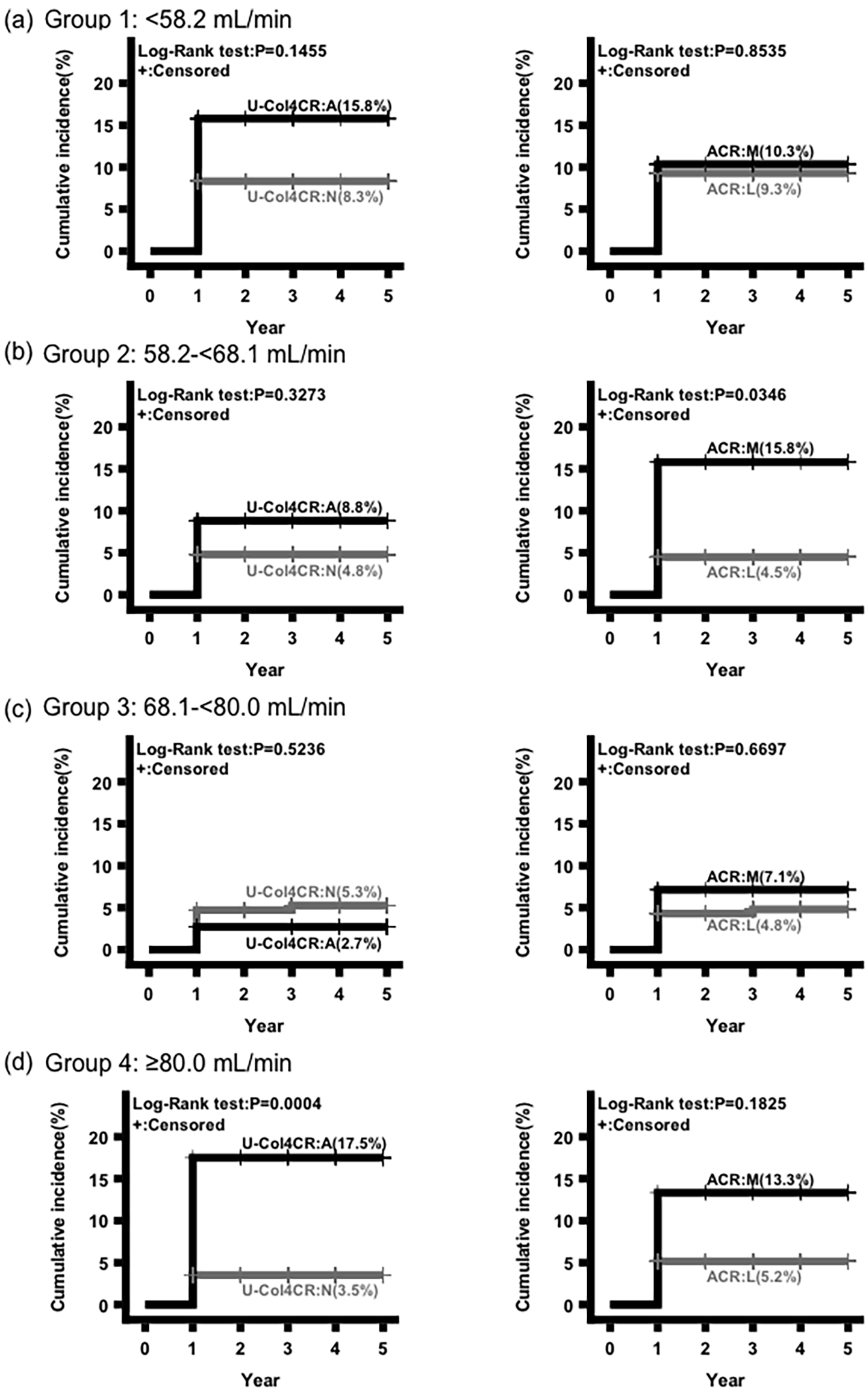
(a) Group 1: <58.2 mL/min
Log-Rank test:P=0.1455
+:Censored
U-Col4CR:A(15.8%)
U-Col4CR:N(8.3%)
Log-Rank test:P=0.8535
+:Censored
ACR:M(10.3%)
ACR:L(9.3%)
(b) Group 2: 58.2-<68.1 mL/min
Log-Rank test:P=0.3273
+:Censored
U-Col4CR:A(8.8%)
U-Col4CR:N(4.8%)
Log-Rank test:P=0.0346
+:Censored
ACR:M(15.8%)
ACR:L(4.5%)
(c) Group 3: 68.1-<80.0 mL/min
Log-Rank test:P=0.5236
+:Censored
U-Col4CR:N(5.3%)
U-Col4CR:A(2.7%)
Log-Rank test:P=0.6697
+:Censored
ACR:M(7.1%)
ACR:L(4.8%)
(d) Group 4: ≥80.0 mL/min
Log-Rank test:P=0.0004
+:Censored
U-Col4CR:A(17.5%)
U-Col4CR:N(3.5%)
Log-Rank test:P=0.1825
+:Censored
ACR:M(13.3%)
ACR:L(5.2%)
Cumulative incidence(%)
Year
0
1
2
3
4
5
10
15
20

Fig 3. Kaplan-Meier curves to evaluate the prognostic value of urinary type IV collagen-creatinine ratio (U-Col4CR) or urinary albumin-creatinine ratio (ACR) for 10% eGFR change per year. A: Abnormal urinary type IV collagen-creatinine ratio. N: Normal urinary type IV collagen-creatinine ratio. M: Microalbuminuria. L: Normoalbuminuria.

https://doi.org/10.1371/journal.pone.0195523.g003

(Table 5). However, albuminuria can be affected by several CKD-related factors inclusive of aging, metabolic syndrome, hypertension, and diabetes, taking the ACR values into account in eGFR groups (Table 1). This hypothesis is supported by previous results of studies on the various causes of albuminuria including hemodynamic, permeability, and histological abnormalities [28–32].

Interestingly, the number of participants with abnormal U-Col4CR was not different among eGFR groups, whereas the number of participants with microalbuminuria was higher in low eGFR groups (Table 1). U-Col4CR was not affected by hypertension and aging compared with ACR. We speculate that some individuals have kidney structural or hemodynamic impairment related to abnormal U-Col4CR, although the cause of kidney impairment is unknown. This hypothesis is consistent with that of a previous report by Furumatsu et al. [7], which indicated that not a few patients with representative nephrotic syndrome and glomerulonephritis had abnormal U-Col4CR. This kidney impairment can appear as a prognostic factor for kidney dysfunction aside from other well-known risk factors such as aging, metabolic syndrome, hypertension and diabetes. The mechanism of abnormal U-Col4 excretion in nondiabetic kidney disease is apparently different from that of albuminuria, but this has not been clarified yet. In our study, 77 participants had microalbuminuria and 149 exhibited abnormal U-Col4CR, but only 28 showed both microalbuminuria and abnormal U-Col4CR (data not shown). In another Japanese cross-sectional study, of the 1079 Japanese general population without diabetes aged 40 to 65 years who had normal urinalysis findings and serum Cr concentration <1.5 mg/dL, 54 had ACR >37 mg/gCr and 54 exhibited U-Col4CR >7.9 μg/gCr. However, only 7 participants showed both abnormal ACR and U-Col4CR [33]. Abnormal U-Col4 excretion in diabetes indicated the development of glomerular matrix accumulation, which has been shown to result in compromised renal filtration function. A study investigating U-Col4 excretion in patients with type 1 or type 2 diabetes showed a significant inverse correlation between the reciprocal of the serum Cr concentration and U-Col4 excretion, irrelevantly to the degree of albuminuria [34]. In addition, Banu et al. reported that U-Col4 excretion was related to not only albuminuria but also to renal tubular damage markers such as urinary N-acetyl-β-D-glucosaminidase and alpha1-microglobulin in patients with type 2 diabetes. [35]. Therefore, unlike albuminuria, abnormal U-Col4 excretion could likely reflect histological kidney damage related to an impaired balance between collagen IV synthesis and degradation in glomeruli and tubules simply, although only a few evidences indicated the association of U-Col4 excretion with pathological kidney damage in human nondiabetic kidney diseases. For instance, Enomoto et al. reported that renovascular stiffness, which is probably due to glomerulosclerosis, was correlated with U-Col4 excretion in patients with essential hypertension [36]. Consequently, we could show that U-Col4CR is one of the possible prognostic factors for the decline in eGFR in the general population without diabetes, independent of albuminuria. We believe that U-Col4CR during a health examination should be measured to reveal an independent hidden risk for the development of kidney dysfunction and help detect future complications such as diabetic kidney disease in every individual.

This study has some limitations. The equation for calculating eGFR is not very accurate for assessing GFR ≥60 ml/min/1.73m^2 [11, 37], and this equation was demonstrated by evaluating the hospitalized patients with CKD. Therefore, it is inappropriate to apply the eGFR equation

to estimate kidney function in the general population [11]. However, using the eGFR equation, a recent large-scale study of the general population has shown that individuals with eGFR ≥60 ml/min/1.73m^2 have a significant risk for developing end-stage kidney disease, if they exhibit more than 30% eGFR reduction in 2 or 3 years. Therefore, the use of the eGFR equation to investigate the risk factors for kidney dysfunction in patients with eGFR ≥60 ml/min/1.73m^2 could be meaningful [9,20]. Furthermore, only a few reports have investigated the prognostic factors for the decline in eGFR in the general population without CKD, probably because of eGFR uncertainty in this population. Therefore, our study can trigger the start of revealing hidden risk factors for kidney dysfunction in the general population with eGFR ≥60 mL/min/1.73m^2 using the eGFR equation.

Conclusions

It is important to measure U-Col4CR even in the general population without diabetes, because abnormal U-Col4CR can be a risk factor for the decline in eGFR in individualis without diabetes and with normal kidney function and urinalysis findings. U-Col4CR measurement can help detect future complications such as diabetic kidney disease.

Supporting information

S1 File. All relevant data derived from participants' annual health examinations. (XLSX)

Acknowledgments

We thank Yoko Okazawa (Tokushima University) for the clinical assistance and Editage (Tokyo, Japan) for English language editing.

Author Contributions

Conceptualization: Yasuhiko Koezuka, Go Ichien.

Data curation: Tatsuya Tominaga, Masanori Tamaki, Eriko Shibata, Taichi Murakami, Seiji Kishi, Hideharu Abe.

Formal analysis: Norimichi Takamatsu, Naoto Minagawa.

Writing – original draft: Fumi Kishi, Kojiro Nagai.

Writing – review & editing: Toshio Doi.

References

1. Matheson A, Willcox MD, Flanagan J, Walsh BJ. Urinary biomarkers involved in type 2 diabetes: a review. Metab Res Rev. 2010; 26:150–171.
2. Kado S, Aoki A, Wada S, Katayama Y, Kugai N, Yoshizawa N, et al. Urinary type IV collagen as a marker for early diabetic nephropathy. Diabetes Res Clin Pract. 1996; 31:103–108. PMID: 8792108
3. Morita M, Uchigata Y, Hanai K, Ogawa Y, Iwamoto Y. Association of urinary type IV collagen with GFR decline in young patients with type 1 diabetes. Am J Kidney Dis. 2011; 58:915–920. https://doi.org/10.1053/j.ajkd.2011.04.019 PMID: 21763044
4. Katavetin P, Katavetin P, Susantitaphong P, Townamchai N, Tiranathanagul K, Tungsanga K, et al. Urinary type IV collagen excretion predicts subsequent declining renal function in type 2 diabetic patients with proteinuria. Diabetes Res Clin Pract. 2010; 89:e33–35. https://doi.org/10.1016/j.diabres.2010.05.007 PMID: 20542586
5. National Kidney Foundation. K/DOQI clinical practice guidelines for chronic kidney disease: evaluation, classification, and stratification. Am J Kidney Dis. 2002; 39(2 Suppl 1):S1–266.

6. Glassock RJ. Is the presence of microalbuminuria a relevant marker of kidney disease? Curr Hypertens Rep. 2010; 12:364–368. https://doi.org/10.1007/s11906-010-0133-3 PMID: 20686930
7. Furumatsu Y, Nagasawa Y, Shoji T, Yamamoto R, Iio K, Matsui I, et al. Urinary type IV collagen in non-diabetic kidney disease. Nephron Clin Pract. 2011; 117:c160–166. https://doi.org/10.1159/000319794 PMID: 20699621
8. Takamatsu N, Abe H, Tominaga T, Nakahara K, Ito Y, Okumoto Y, et al. Risk factors for chronic kidney disease in Japan: a community-based study. BMC Nephrol. 2009; 10:34. https://doi.org/10.1186/1471-2369-10-34 PMID: 19860890
9. Coresh J, Turin TC, Matsushita K, Sang Y, Ballew SH, Appel LJ, et al. Decline in estimated glomerular filtration rate and subsequent risk of end-stage renal disease and mortality. JAMA. 2014; 311:2518–2531. https://doi.org/10.1001/jama.2014.6634 PMID: 24892770
10. Ando Y, Minami H, Saka H, Ando M, Sakai S, Shimokata K. Adjustment of creatinine clearance improves accuracy of Calvert's formula for carboplatin dosing. Br J Cancer. 1997; 76:1067–1071. PMID: 9376268
11. Matsuo S, Imai E, Horio M, Yasuda Y, Tomita K, Nitta K, et al. Revised equations for estimated GFR from serum creatinine in Japan. Am J Kidney Dis. 2009; 53:982–992. https://doi.org/10.1053/j.ajkd.2008.12.034 PMID: 19339088
12. DuBois D, DuBois EF. A formula to estimate the approximate surface area if height and weight be known. Arch Intern Med. 1916; 17:863–871.
13. Kashiwagi A, Kasuga M, Araki E, Oka Y, Hanafusa T, Ito H, et al. International clinical harmonization of glycated hemoglobin in Japan: From Japan Diabetes Society to National Glycohemoglobin Standardization Program values. J Diabetes Invest. 2012; 3:39–40.
14. American Diabetes Association Clinical Practice Recommendations 2001. Diabetes Care. 2001; 24: S1–133. PMID: 11403001
15. Araki S, Haneda M, Koya D, Isshiki K, Kume S, Sugimoto T, et al. Association between urinary type IV collagen level and deterioration of renal function in type 2 diabetic patients without overt proteinuria. Diabetes Care. 2010; 33:1805–1810. https://doi.org/10.2337/dc10-0199 PMID: 20668153
16. Obata K, Iwata K, Ichida T, Inoue K, Matsumoto E, Muragaki Y, et al. One step sandwich enzyme immunoassay for human type IV collagen using monoclonal antibodies. Clin Chim Acta. 1989; 181:293–303. PMID: 2547537
17. Hirayama A, Konta T, Kamei K, Suzuki K, Ichikawa K, Fujimoto S, et al. Blood pressure, proteinuria, and renal function decline: associations in a large community-based population. Am J Hypertens. 2015; 28:1150–1156. https://doi.org/10.1093/ajh/hpv003 PMID: 25673040
18. Matsumoto A, Nagasawa Y, Yamamoto R, Shinzawa M, Hasuike Y, Kuragano T, et al. The association of alcohol and smoking with CKD in a Japanese nationwide cross-sectional survey. Hypertens Res. 2017; 40:771–778. https://doi.org/10.1038/hr.2017.25 PMID: 28275237
19. Iseki K, Asahi K, Moriyama T, Yamagata K, Tsuruya K, Yoshida H, et al. Risk factor profiles based on estimated glomerular filtration rate and dipstick proteinuria among participants of the Specific Health Check and Guidance System in Japan 2008. Clin Exp Nephrol. 2012; 16:244–249. https://doi.org/10.1007/s10157-011-0551-9 PMID: 22057582
20. Imai E, Horio M, Yamagata K, Iseki K, Hara S, Ura N, et al. Slower decline of glomerular filtration rate in the Japanese general population: a longitudinal 10-year follow-up study. Hypertens Res. 2008; 31:433–441. https://doi.org/10.1291/hypres.31.433 PMID: 18497462
21. Kanda E, Usui T, Kashihara N, Iseki C, Iseki K, Nangaku M. Importance of glomerular filtration rate change as surrogate endpoint for the future incidence of end-stage renal disease in general Japanese population: community-based cohort study. Clin Exp Nephrol. 2017 Sep 7. https://doi.org/10.1007/s10157-017-1463-0 PMID: 28884361
22. Chang WX, Asakawa S, Toyoki D, Nemoto Y, Morimoto C, Tamura Y, et al. Predictors and the subsequent risk of end-stage renal disease—usefulness of 30% decline in estimated GFR over 2 years. PLoS ONE. 2015; 10:e0132927. https://doi.org/10.1371/journal.pone.0132927 PMID: 26177463
23. Levey AS, Inker LA, Matsushita K, Greene T, Willis K, Lewis E, et al. GFR decline as an end point for clinical trials in CKD: a scientific workshop sponsored by the National Kidney Foundation and the US Food and Drug Administration. Am J Kidney Dis. 2014; 64:821–835. https://doi.org/10.1053/j.ajkd.2014.07.030 PMID: 25441437
24. Matsushita K, Chen J, Sang Y, Ballew SH, Shimazaki R, Fukagawa M, et al. Risk of end-stage renal disease in Japanese patients with chronic kidney disease increases proportionately to decline in estimated glomerular filtration rate. Kidney Int. 2016; 90:1109–1114. https://doi.org/10.1016/j.kint.2016.08.003 PMID: 27666758

25. Hanevold CD, Pollock JS, Harshfield GA. Racial differences in microalbumin excretion in healthy adolescents. Hypertension. 2008; 51:334–338. https://doi.org/10.1161/HYPERTENSIONAHA.107.098095 PMID: 18172060
26. Trachtenberg F, Barregård L. The effect of age, sex, and race on urinary markers of kidney damage in children. Am J Kidney Dis. 2007; 50:938–945. https://doi.org/10.1053/j.ajkd.2007.08.014 PMID: 18037094
27. National Kidney Foundation. K/DOQI clinical practice guidelines for chronic kidney disease: evaluation, classification, and stratification. Am J Kidney Dis. 2002; 39:S1–266. PMID: 11904577
28. Tian X, Ishibe S. Targeting the podocyte cytoskeleton: from pathogenesis to therapy in proteinuric kidney disease. Nephrol Dial Transplant. 2016; 31:1577–1583. https://doi.org/10.1093/ndt/gfw021 PMID: 26968197
29. Suh JH, Miner JH. The glomerular basement membrane as a barrier to albumin. Nat Rev Nephrol. 2013; 9:470–477. https://doi.org/10.1038/nrneph.2013.109 PMID: 23774818
30. Dickson LE, Wagner MC, Sandoval RM, Molitoris BA. The proximal tubule and albuminuria: really! J Am Soc Nephrol. 2014; 25:443–453. https://doi.org/10.1681/ASN.2013090950 PMID: 24408874
31. Locatelli F, Pozzoni P, Del Vecchio L. Renal manifestations in the metabolic syndrome. J Am Soc Nephrol. 2006; 17:S81–85. https://doi.org/10.1681/ASN.2005121332 PMID: 16565254
32. Mulè G, Castiglia A, Cusumano C, Scaduto E, Geraci G, Altieri D, et al. Subclinical kidney damage in hypertensive patients: a renal window opened on the cardiovascular system. Focus on microalbuminuria. Adv Exp Med Biol. 2017; 956:279–306. https://doi.org/10.1007/5584_2016_85 PMID: 27873229
33. Ishimitsu T, Murayama N, Meguro T, Kageyama Y, Okada K, Okubo Y, et al. Urinary excretions of albumin and type IV collagen in normotensive and hypertensive subjects. Hypertens Res. 2000; 23:459–466. PMID: 11016800
34. Cohen MP, Lautenslager GT, Shearman CW. Increased collagen IV excretion in diabetes. A marker of compromised filtration function. Diabetes Care. 2001; 24:914–918. PMID: 11347754
35. Banu N, Hara H, Okamura M, Egusa G, Yamakido M. Urinary excretion of type IV collagen and laminin in the evaluation of nephropathy in NIDDM: comparison with urinary albumin and markers of tubular dysfunction and/or damage. Diabetes Res Clin Pract. 1995; 29:57–67. PMID: 8593760
36. Enomoto D, Okura T, Nagao T, Jotoku M, Irita J, Miyoshi K, et al. Relationship between renal hemodynamics and urinary type IV collagen in patients with essential hypertension. Clin Exp Hypertens. 2012; 34:612–616. https://doi.org/10.3109/10641963.2012.683911 PMID: 22564177
37. Glassock RJ, Winearls C. Ageing and the glomerular filtration rate: truths and consequences. Trans Am Clin Climatol Assoc. 2009; 120:419–428. PMID: 19768194
